# Synthesis of Selenium-Containing Polystyrene Microspheres and Using as Catalyst for Oxidation of Acrolein

**DOI:** 10.3390/polym13101632

**Published:** 2021-05-18

**Authors:** Yuanyuan Zhang, Xiangqiang Pan, Jian Zhu

**Affiliations:** State and Local Joint Engineering Laboratory for Novel Functional Polymeric Materials, Jiangsu Key Laboratory of Advanced Functional Polymer Design and Application, Department of Polymer Science and Engineering, College of Chemistry, Chemical Engineering and Materials Science, Soochow University, Suzhou 215123, China; 15062993300@163.com

**Keywords:** polystyrene, microspheres, catalyst

## Abstract

Selenium-containing polystyrene (DSe-PS) microspheres were synthesized by soap-free emulsion polymerization using 1,2-bis(2,3,5,6-tetrafluoro-4-vinylphenyl)diselane (FVPDSe) and divinylbenzene (DVB) as crosslinking agents. The particle size of the obtained DSe-PS was characterized by a scanning electron microscope and dynamic light scattering. The results showed that the diameter of the obtained DSe-PS microspheres could be adjusted by changing the ratio of the monomer and crosslinker/water. The diselenide moiety in the obtained DSe-PS microspheres could be oxidized to seleninic acid by H_2_O_2_ which can catalyze the oxidation of acrolein. The oxidized DSe-PS microspheres exhibited higher catalytic activity and selectivity to methyl acrylate in a model oxidation of acrolein.

## 1. Introduction

Selenium was first discovered by the Swedish chemist Jons Jakob Berzelius in the red residue from a sulfuric acid plant in 1817 [1]. In 1957, selenium was discovered to be a trace element needed by humans [2] and appropriate intake of selenium is beneficial to human health. In addition, selenium is now gradually becoming transformed into an environmentally friendly tool. In 1973, glutathione peroxidase was discovered as the first selenium-containing enzyme [3]. This is an enzyme that protects the human body from peroxidation damage and catalyzes the decomposition of peroxides, and is of great significance for maintaining the redox balance of the body and maintaining the stability of the internal environment.

Organic selenium compounds are limited in their promotion and application due to unpleasant odor, high toxicity and low stability. In contrast, the development of selenium-containing polymers partially reduced these problems. In 1969, Zundel reported the application of polymer-supported organic selenium compounds in organic synthesis [4]. The method of immobilizing the selenide on the polymer not only improves the stability of the selenium compound, but also increases the possibility of recycling the selenium compound. In recent years, the synthesis and application of selenium-containing polymers have gradually attracted the attention of researchers [5]. Selenium-containing polymers can be used to produce polymer materials with a high refractive index for the high molar refraction of selenium-containing groups [6,7]. Selenium compounds have a variety of valence states and are easily oxidized or reduced, so they can be used to prepare functional materials with redox responsiveness [8,9]. Se-Te, Se-Se, Se-S and Se-N have weak bond energy and can be used to prepare self-healing materials [10], shape memory materials [11], etc. Selenium-containing polymers are also used as adsorbents for metal elements [12,13] or to synthesize quantum dots (CdSe) [14] based on the interaction between metal and selenium. In addition, selenium-containing compounds are also widely used in synthesizing catalysts [15]. Selenium-based catalysts have been proved to be wonderful candidates for selectively oxidative reactions of various compounds.

On the other hand, monodisperse polymer microspheres have attracted great attention due to their excellent properties such as strong adsorption, large specific surface area and strong surface reactivity. Monodisperse polymer microsphere material refers to a polymer material that has a spherical structure and is uniform in size. The polymer microspheres can be obtained by emulsion polymerization [16], dispersion polymerization [17], suspension polymerization [18] and precipitation polymerization [19]. It has a wide range of applications in biomedicine, standard metering, chromatographic packing, catalyst carriers, adsorbents, etc.

Organic selenium catalysts exhibit excellent catalytic performance in many important organic reactions, such as oxidation reaction [20], halogenation reaction [21], cyclization reaction [22] and ring expansion reaction [23]. In recent years, the number of reports on heterogeneous catalysis systems containing selenium has gradually increased. Since 1999, Detty and coworkers had been committed to the study of selenium-containing polymers to catalyze the bromination of organic substrates. They developed dendritic polyphenylselenides and gels chelating selenium oxides and successfully applied them to the bromination of organic substrates [24,25]. In 2006, Barrero and partners used polymer-supported selenenyl bromide to catalyze the regioselective halogenation of the terminal isopropylidene unit of different acyclic polyolefinic polyprenoids [26]. In 2018, Nemati and co-authors loaded a diselenide catalyst on silica-coated Fe_3_O_4_ nanoparticles to prepare a magnetic recyclable material that was used to catalyze the oxidation of aldehydes into carboxylic acids [27]. In 2019, Pich et al. produced a microgel containing diselenium that selectively catalyzes acrolein into acrylic acid and methyl acrylate [28].

Based on the above literature research, we can see that although organic selenium compounds have excellent catalytic performance, there are relatively few studies on heterogeneous catalytic systems containing selenium. Herein, we combined polymer microspheres and the functionality of selenium-containing compounds to prepare selenium-containing polystyrene microspheres and their use as heterogeneous catalysts.

## 2. Materials and Methods

**Scheme 1:**
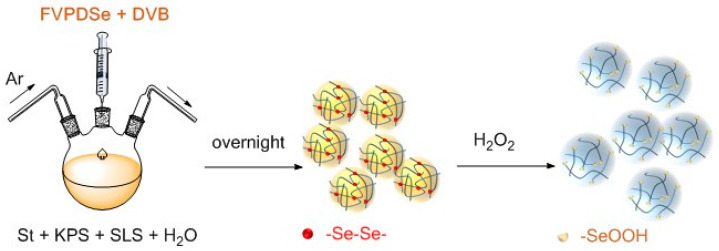
Selenium-containing polystyrene (DSe-PS) microspheres were synthesized by soap-free emulsion polymerization. The DSe-PS can be oxidized by H_2_O_2_, making the diselenide bond into seleninic acid.

In this article, narrow-disperse selenium-containing polystyrene microspheres were synthesized by soap-free emulsion polymerization using styrene as a monomer, divinylbenzene as a crosslinking agent and 1,2-bis(2,3,5,6-tetrafluoro-4-vinylphenyl)diselane as a selenium source and another crosslinking agent. Subsequently, H_2_O_2_ was used to oxidize the diselenide bond in the microspheres to form seleninic acid, which could be used as a heterogeneous catalyst to catalyze the oxidation of acrolein (Scheme 1). The microsphere structure provided a catalyst with possible recyclability and development potential in catalyzing the oxidation reaction of unsaturated aldehydes.

### 2.1. Materials

Styrene (St) and divinylbenzene (DVB) were purchased from Sinopharm Group Chemical Reagent Co., Ltd. (Shanghai China) and purified by passing them through a short neutral aluminum oxide column before use. Potassium persulfate (K_2_S_2_O_8_, KPS), sodium borohydride (NaBH_4_), sodium hydroxide (NaOH) and hydrogen peroxide (30% aqueous solution, H_2_O_2_) were purchased from Sinopharm Chemical Reagent Co., Ltd. Shanghai China and used as received. 2,3,4,5,6-Pentafluorostyrene was purchased from J&K and used as received. Bromopentafluorobenzene was purchased from TCI and used as received.

### 2.2. Characterization

^1^H NMR and ^13^C NMR spectra were collected with a Bruker nuclear magnetic resonance instrument (300 MHz) using tetramethylsilane (TMS) as the internal standard at room temperature. ^77^Se NMR spectra were measured on an Agilent Technologies spectrometer (600 MHz). Morphology of the sample was characterized via a Hitachi SU8010 scanning electron microscope (SEM) with an operation voltage of 5 kV. The particle size of the sample was characterized by a Brookhaven dynamic light scattering (DLS) nanoparticle size analyzer. The conversion rate of the catalytic reaction was calculated with the characterization data of Varian cp3800 gas chromatography. X-ray photoelectron spectroscopy (XPS) was carried out on the Thermo Fisher Scientific ESCALAB 250 XI, with Al KR source to confirm the change in the chemical structure.

### 2.3. Synthesis of 1, 2-bis(2,3,5,6-tetrafluoro-4-vinylphenyl)diselane (FVPDSe)

**Scheme 2:**
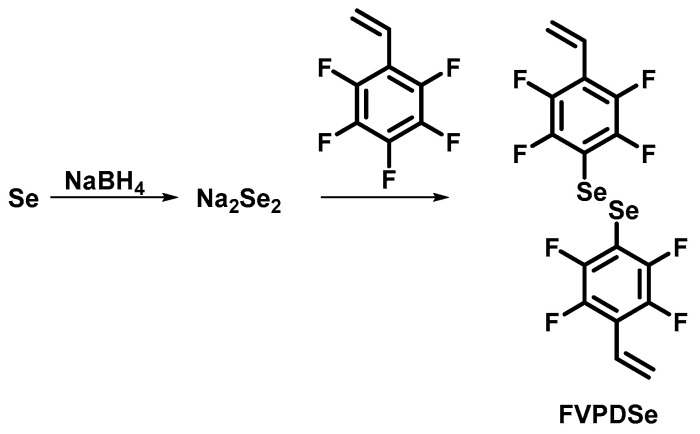
Synthesis of 1, 2-bis(2,3,5,6-tetrafluoro-4-vinylphenyl)diselane.

FVPDSe was synthesized according to the literature [29] with optimized conditions (Scheme 2). NaOH (2.00 g, 50 mmol) was dissolved in 20 mL deionized water, then selenium powder (3.95 g, 50 mmol) was added and NaBH_4_ (0.24 g, 6.3 mmol) in batches with stirring until the solution finally turned reddish brown. Then, 2,3,4,5,6-pentafluorostyrene (9.70 g, 50 mmol) dissolved in 50 mL *N*,*N*-dimethylformamide (DMF) was added dropwise in situ, and stirred overnight at room temperature. Argon was used throughout the whole procedure to maintain an inert atmosphere. Subsequently, NaBH_4_ (0.25 g, 6.6 mmol) was added. After extracting with EA, the water phase was adjusted to acidity by adding hydrochloric acid, then air was blown for coupling. The product was obtained after fast column chromatography with petroleum ether.

^1^H NMR (CDCl_3_, 300 MHz) δ 6.73 (dd, 1H), 6.18 (d, 1H), 5.78 (d, 1H).

^13^C NMR (CDCl_3_, 75 MHz) δ 147.67 (ddt), 144.83 (ddt), 125.64 (t), 122.95, 119.88 (t), 107.40 (t).

^77^Se NMR (CDCl_3_, 76 MHz,) δ 374.38 (t).

^19^F NMR (CDCl_3_, 376 MHz) δ -127.78 (dd), -142.12 (dd).

### 2.4. Synthesis of Pentafluorophenyl Seleninic Acid

**Scheme 3:**
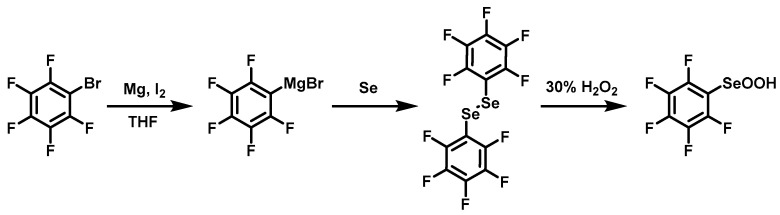
Synthesis of pentafluorophenyl seleninic acid.

1,2-Bis(perfluorophenyl)diselane was synthesized according to the literature [30] with optimized conditions (Scheme 3). Magnesium chips (0.30 g, 12.5 mmol) and a pill of I_2_ were mixed in 16 mL of tetrahydrofuran (THF). Bromopentafluorobenzene (2 g, 8.1 mmol) was added dropwise followed by vigorous stirring for one hour. Finally, selenium powder (0.70 g, 8.8 mmol) was added and stirred vigorously for another hour. Excess magnesium chips were quenched with dilute hydrochloric acid. The product 1,2-bis(perfluorophenyl)diselane was obtained by flash column chromatography with petroleum ether. Pentafluorophenyl seleninic acid was obtained by oxidizing 1,2-bis(perfluorophenyl)diselane with H_2_O_2_ (30%) in dichloromethane.

^1^H NMR (DMSO, 300 MHz) δ 5.58 (s, 1H).

### 2.5. General Procedure for the Preparation of Diselenide-Containing Polystyrene (DSe-PS) Spheres

**Scheme 4:**
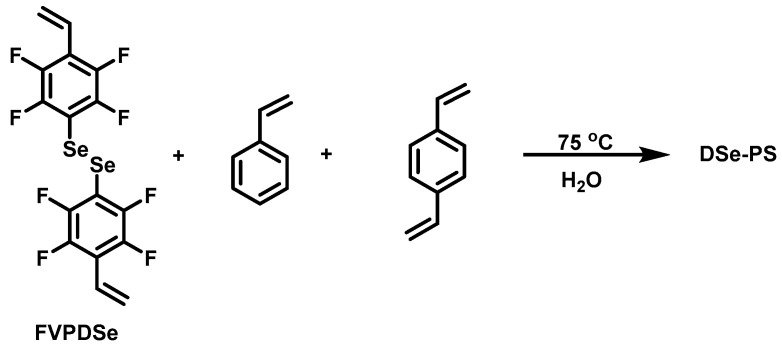
Preparation route of DSe-PS.

Series of DSe-PS were synthesized by soap-free polymerization (Scheme 4). SLS (0.01 g, 0.036 mmol) was dissolved in 40 mL deionized water with stirring for 30 min. St (1.0 g, 9.6 mmol) and KPS (0.04 g, 0.15 mmol) were added into the solution followed by stirring for 30 min under argon atmosphere. The mixture was heated to 75 °C for the polymerization. After stirring for three hours, degassed DVB and FVPDSe were added as double crosslinking agents, and stirred for another 12 h. DSe-PS was obtained after centrifugation.

### 2.6. Oxidation Reactions

DSe-PS in methanol was oxidized by 5% H_2_O_2_ at room temperature for 24 h. The oxidized DSe-PS, denoted as Se-PS, was obtained after centrifugation. Ten micrograms of oxidized DSe-PS dispersed in 5 mL methanol was mixed with acrolein (0.56 g, 0.01 mmol), 1.3 mL H_2_O_2_ (30%) and a bit of hydroquinone as an inhibitor, then kept at 50 °C overnight and the conversion rate was analyzed by gas chromatography.

## 3. Results and Discussion

Selenium functionalized compounds, especially diselenides, have been widely used as oxidative catalysts due to their responsive redox behavior [15]. Herein, we introduced diselenide functional groups into a polymer chain which offered the possibility of reuse. For this, diselenide functionalized monodisperse polystyrene (DSe-PS) was synthesized by soap-free emulsion polymerization of styrene using FVPDSe and DVB as the crosslinkers. After the DSe-PS was oxidized by hydrogen peroxide, Se-PS containing seleninic acid was formed to catalyze the oxidation of acrolein.

**Figure 1:**
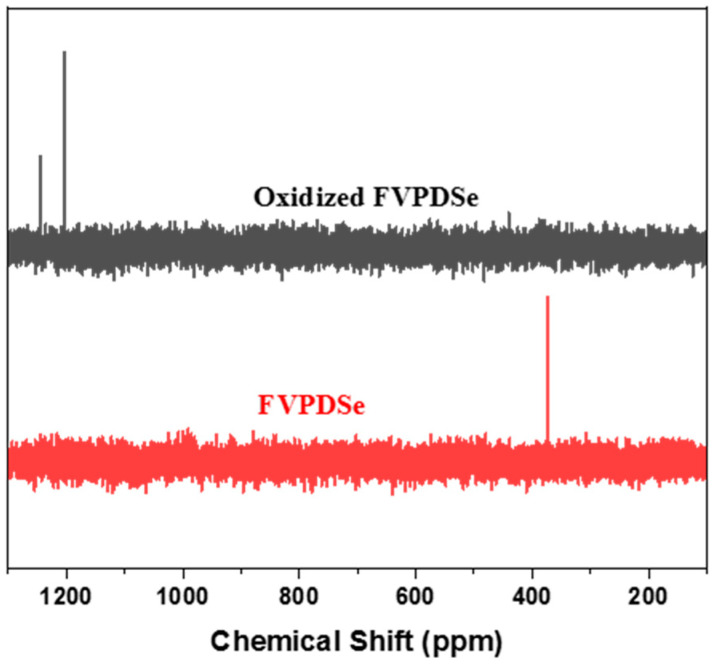
^77^Se NMR spectra of FVPDSe before and after oxidation (red-before oxidation; black-after oxidation).

Firstly, the oxidation behavior of the diselenide bond was investigated by using FVPDSe as a small molecule model. The ^77^Se NMR spectrum of FVPDSe was characterized before and after oxidation. The results are summarized in Figure 1. It shows that the selenium signal at δ = 373 ppm in deiselenide was shifted to 1204 ppm which, corresponding to seleninic acid after oxidation by excessive H_2_O_2_ (30 times that of FVPDSe) at room temperature. The chemical shift at δ = 1244 ppm could be from a small amount of a selenium derivative with a higher oxidation state. Such results indicated the successful oxidization of diselenide in FVPDSe under mild conditions. The formation of an oxidized selenium structure could be used to selectively oxidize organic compounds [28].

**Figure 2:**
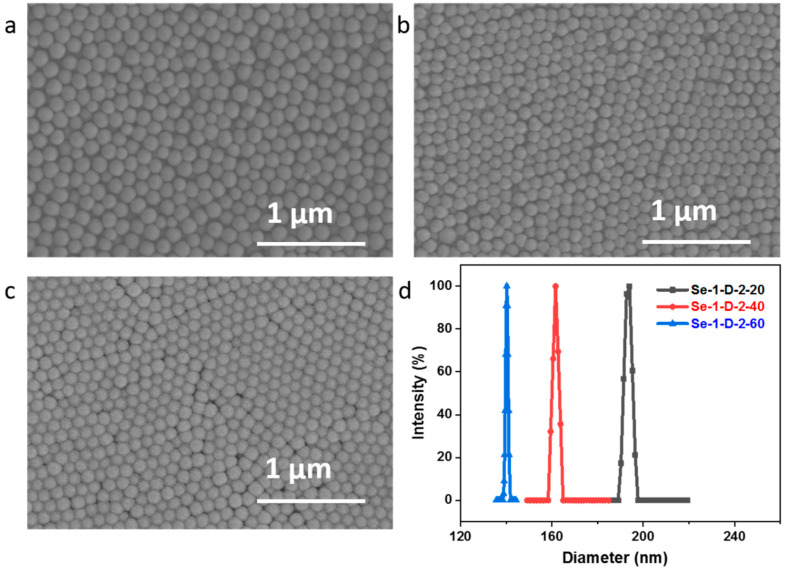
SEM images of DSe-PS. (**a**) Se-1-D-2-20, (**b**) Se-1-D-2-40, (**c**) Se-1-D-2-60. (**d**) Particle size distribution curve of DSe-PS microspheres, polydispersity indexes (PDI) were a = 0.1, b = 0.155, c = 0.013.

Then, a diselenide group was introduced into polymer particles by using FVPDSe as the crosslinker in soap-free emulsion polymerization of DSe-PS in an aqueous medium at 75 °C. The polymerizations were carried out by using different amounts of crosslinker. Two crosslinker, e.g., FVPDSe and DVB, were used in preparing polymer particles for the consideration of possible fragmentation of diselenide in FVPDSe after oxidization. The results are summarized in Table 1. It shows that polymer particles could be adjusted by varying the concentration of crosslinker and the amount of water. Polymeric particles with a dimeter of 141–190 nm could be obtained in these conditions. The morphology of the obtained particles was characterized by SEM, as shown in Figure 2a–c. The results showed that regular spherical particles were obtained after polymerization. The particle size almost showed monodispersity, as identified by DLS analysis (Figure 2d).

**Figure 3:**
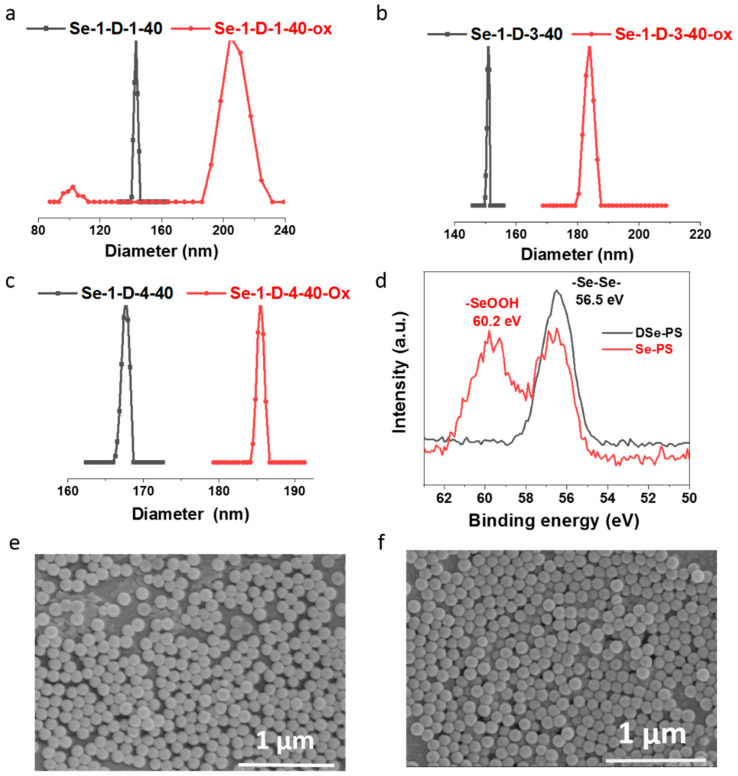
Particle size distribution curve of DSe-PS before and after oxidation by H_2_O_2_ (black-before oxidation; red-after oxidation). (**a**) Se-1-D-1-40, (**b**) Se-1-D-3-40, (**c**) Se-1-D-4-40. (**d**) XPS spectra of Se in DSe-PS and Se-PS which was oxidized by 5 wt% H_2_O_2_ overnight. SEM images of Se-1-D-3-40 (**e**) before and (**f**) after oxidation.

After obtaining these polymer particles, the oxidization of diselenide in DSe-PS was investigated. Polystyrene microspheres with different selenium contents (mass ratio of DVB:FVPDSe = 1:1, 2:1, 3:1, 4:1) were oxidized by 5 wt% H_2_O_2_ overnight at room temperature. The polystyrene microspheres before and after oxidation were characterized by DLS, XPS and SEM. The results showed in Figure 3 indicated that the morphology of particles remained as a regular sphere after oxidization. However, the size of the microspheres increased after oxidation, e.g., from 148 nm to 198 nm with DVB:FVPDSe = 1:1, 149 nm to 186 nm with DVB:FVPDSe = 3:1 and 167 nm to 188 nm with DVB:FVPDSe = 4:1, while the Se3d XPS spectra in Figure 3d show the binding energy of diselenide at 56.5 eV, and the binding energy of Se3d shifted to 60.2 eV which corresponds to seleninic acid after oxidation [31]. We speculated that the diselenide bond in DSe-PS is oxidized to form seleninic acid after being oxidized by H_2_O_2_, and becomes porous and loose, which leads to an increase in the measured particle size in methanol. At the same time, the morphology of the sphere did not change after oxidation, and the microspheres could be recycled and reused, which presents the possibility of DSe-PS as a heterogeneous catalyst.

**Scheme 5:**
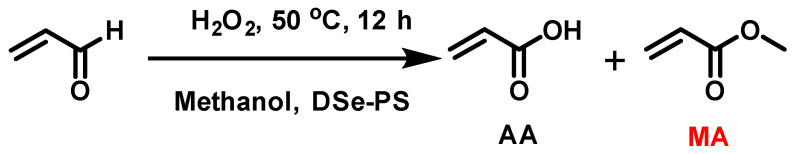
Oxidation of acrolein by H_2_O_2_ catalyzed by DSe-PS in methanol.

To evaluate the catalytic activity of DSe-PS, the oxidation of acrolein was used as a model reaction (Scheme 5). The oxidation of acrolein under the catalysis of selenium-containing compounds has been covered well in the literature [28,32]. Two main products, e.g., acrylic acid (AA) and methacrylate (MA), were obtained after oxidation. It was found that the molar fraction of MA in the products varied from 0.3–0.5 to 0.9–0.95 when using different selenium-based catalysts. A molar fraction of MA in the products is 0.3–0.5 in the oxidation of acrolein when H_2_SeO_3_ is used as a catalyst [32], while by introducing diselenide moiety into microgels and using such selenium-modified microgels as the catalyst, the molar fraction of MA in the products could be increased to 0.9–0.95 [28]. Following such results, DSe-PS was used as the catalyst in the same oxidative reaction. The result showed that the molar fraction of MA in the products was 1.0, i.e., the reaction offered high selective activity for methyl acrylate (Table 2).

**Figure 4:**
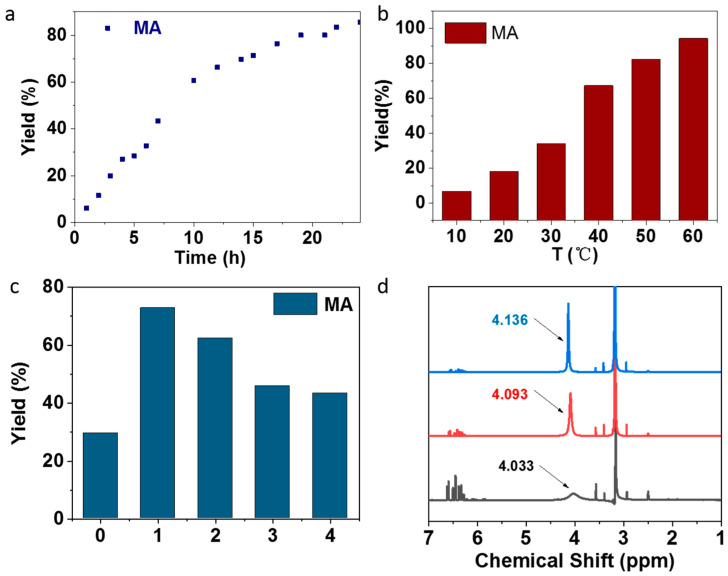
(**a**) Kinetics of the oxidation of acrolein by H_2_O_2_ in methanol catalyzed by oxidized Se-1-D-5-40 (Table S1 in the Supporting Information) at 50 °C for 12 h. (**b**) Oxidation of acrolein catalyzed by oxidized Se-2-D-1-40 (Table S1 in the Supporting Information) at different temperatures for 12 h. (**c**) Oxidation of acrolein catalyzed by the series of oxidized selenium-containing DSe-PS. (0: no catalyst; 1: Se-1-D-1-40, 2: Se-1-D-2-40, 3: Se-1-D-3-40, 4: Se-1-D-4-40). (**d**) ^1^H NMR spectra of pentafluorophenyl seleninic acid and different amounts of methanol in DMSO-*d*_6_ (blue-0.01 g pentafluorophenyl seleninic acid and 0.21 mL methanol, red-0.01 g pentafluorophenyl seleninic acid and 0.11 mL methanol, black-0.01 g pentafluorophenyl seleninic acid and 0.01 mL methanol).

With these initial results, the reaction was investigated in detail. Firstly, the kinetic of reaction was investigated by using oxidized Se-1-D-5-40 as the catalyst at 50 °C. The results are summarized in Figure 4a. The reaction was carried out smoothly and reached over 80% yield of MA after 24 h. Secondly, the effect of reaction temperature on the yields of MA was investigated. The results are summarized in Figure 4b. They showed that the yield of MA was increased with the increasing of temperature. The highest yield of MA was obtained as 94% at 60 °C. Thirdly, the effect of selenium concentration in PS particles on the reaction was investigated. The results are shown in Figure 4c. The maximum yield of MA was 73% in the case of catalyzing by DSe-PS prepared with a weight ratio of FVPDSe and DVB of 1:1. Decreasing the selenium content in DSe-PS resulted in decreasing yields of MA from 73% to 45%. It should be noted here that only MA was obtained in the oxidation of acrolein catalyzed by DSe-PS. We speculated that the hydrogen bond between the fluorine atoms contained in the microspheres and the active hydrogen in methanol promote the rapid esterification of acrylic acid which offered the highly selective formation of MA. In order to verify this hypothesis, the interaction between pentafluorophenyl seleninic acid and methanol was monitored by ^1^H NMR. The results are shown in Figure 4d. They indicated that the chemical shift of protons in methanol was shifted from the original position after cooperation with pentafluorophenyl seleninic acid, which proved the existence of the hydrogen bond. In the case of mixing 0.01 g pentafluorophenyl seleninic acid with 0.01 mL methanol, the signal transformed from a sharp peak to a broad peak due to the formation and fast exchanging of hydrogen bonds.

## 4. Conclusions

In summary, narrow-dispersed selenium-containing polystyrene (DSe-PS) microspheres were synthesized through soap-free emulsion polymerization of styrene using FVPDSe and DVB as dual crosslinking agents. Microspheres with a spherical structure could be obtained. The size of microspheres could be adjusted in the range of 141–190 nm. Although the range showed limitations, it provided the possibility for the controlling of the microspheres’ size. The obtained DSe-PS could be used as an efficient catalyst after oxidation in the oxidative reaction of acrolein, with the highly selective formation of MA reaching the highest yield of 94%. The catalytic reaction had extremely high selectivity towards methyl acrylate for introducing the fluorine atoms into the microspheres. We believe that this material has a promising future in catalyzing the oxidation behavior of unsaturated aldehydes.

## Figures and Tables

**Table 1:** A series of selenium-containing DSe-PS microspheres with different particle sizes or diselenide content.

Sample	St (g)	FVPDSe (g)	DVB (g)	KPS (g)	SLS (g)	H_2_O (mL)	Particle Size (nm) ^1^
Se-1-D-2-20	1.0	0.066	0.132	0.02	0.005	20	190
Se-1-D-2-40	1.0	0.066	0.132	0.04	0.01	40	166
Se-1-D-2-60	1.0	0.066	0.132	0.04	0.01	60	141
Se-1-D-1-40	1.0	0.100	0.100	0.04	0.01	40	148
Se-1-D-3-40	1.0	0.05	0.15	0.04	0.01	40	149
Se-1-D-4-40	1.0	0.04	0.16	0.04	0.01	40	167

^1^ determined by DLS.

**Table 2:** Comparation of the product selectivity of selenium-containing catalysts for the oxidation of acrolein in the literature.

Catalyst	Molar Fraction of MA in the Products
H_2_SeO_3_ [32]	0.3–0.5
Selenium-Modified Microgels [28]	0.9–0.95
DSe-PS	1.0 ^1^

^1^ The molar fraction of MA of oxidation of acrolein with DSe-PS was derived from no acrylic acid peak in the GC traces of oxidation of acrolein catalyzed by DSe-PS (Figures S1–S4 in the Supporting Information).

## Supplementary Materials

The following are available online at https://www.mdpi.com/article/10.3390/polym13101632/s1. Figure S1: The GC traces of acrolein, Figure S2. The GC traces of acrylic acid, Figure S3. The GC traces of methyl acrylate, Figure S4. The GC traces of oxidation of acrolein catalyzed by Se-1-D-5-40, Table S1: A series of selenium-containing DSe-PS.
