# Supplementary material for: Synthesis of Selenium-Containing Polystyrene Microspheres and Using as Catalyst for Oxidation of Acrolein
Source: Polymers (Basel). 2021 May 18;13(10):1632. doi: 10.3390/polym13101632 (PMC8157269; doi:10.3390/polym13101632)
Supplement: Supplementary file 1 [file polymers-13-01632-s001.zip › polymers-1228271-supplementary.pdf]

polymers

Supplementary Materials

Synthesis of Selenium-Containing Polystyrene Microspheres and Useing as Catalyst for Oxidation of Acrolein

Yuanyuan Zhang, Xiangqiang Pan * and Jian Zhu *

State and Local Joint Engineering Laboratory for Novel Functional Polymeric Materials, Jiangsu Key Laboratory of Advanced Functional Polymer Design and Application, Department of Polymer Science and Engineering, College of Chemistry, Chemical Engineering and Materials Science, Soochow University, Suzhou, 215123, China; 15062993300@163.com

* Correspondence: panxq@suda.edu.cn (X.P.); chemzhujian@suda.edu.cn (J.Z.); Tel.: +86-512-6588-3343 (X.P.); +86-512-6588-0726 (J.Z.)

Table S1. A series of selenium-containing DSe-PS.

Sample	St (g)	FVPDSe (g)	DVB (g)	KPS (g)	SLS (g)	H_2O (mL)
Se-1-D-2-20	1.0	0.066	0.132	0.02	0.005	20
Se-1-D-2-40	1.0	0.066	0.132	0.04	0.01	40
Se-1-D-2-60	1.0	0.066	0.132	0.04	0.01	60
Se-1-D-1-40	1.0	0.100	0.100	0.04	0.01	40
Se-1-D-3-40	1.0	0.05	0.15	0.04	0.01	40
Se-1-D-4-40	1.0	0.04	0.16	0.04	0.01	40
Se-1-D-5-40	1.0	0.1	0.5	0.04	0.01	40
Se-2-D-1-40	1.0	0.2	0.1	0.04	0.01	40

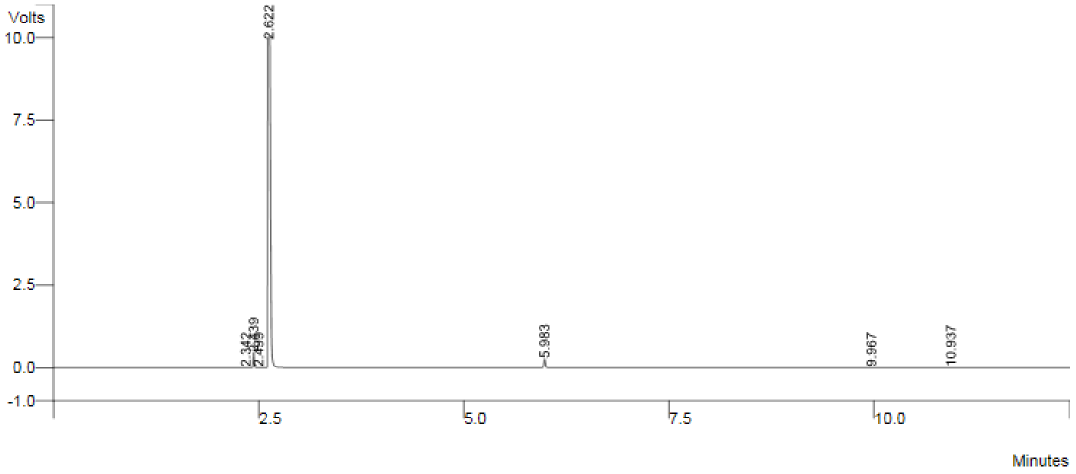

Figure S1. The GC traces of acrolein.

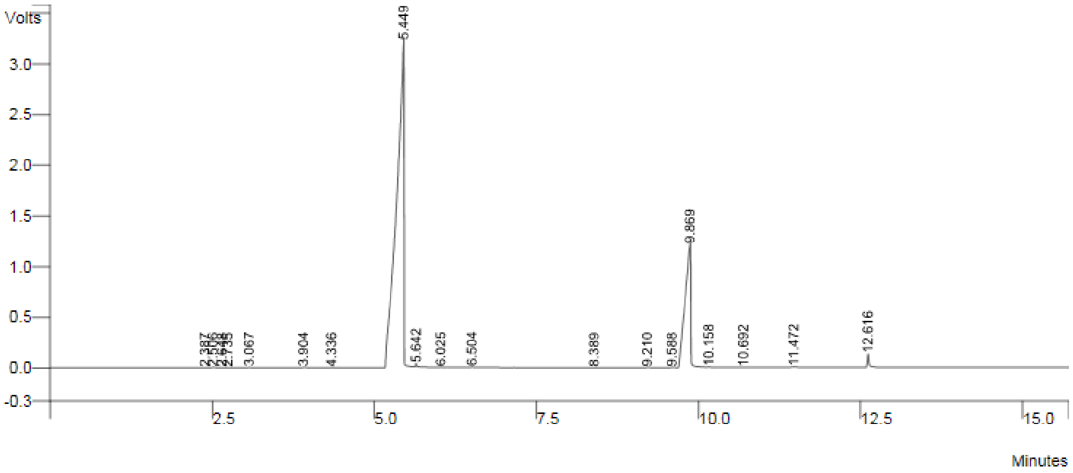

Figure S2. The GC traces of acrylic acid.

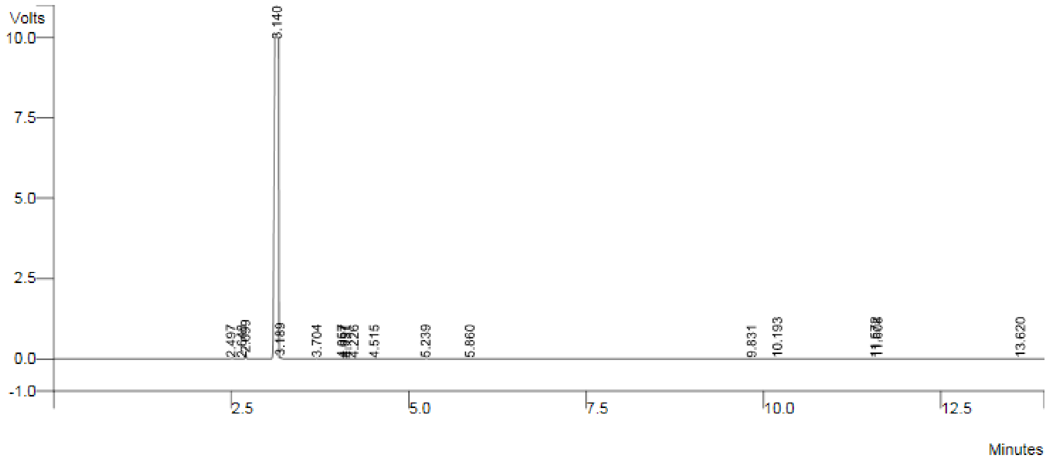

Figure S3. The GC traces of Methyl acrylate.

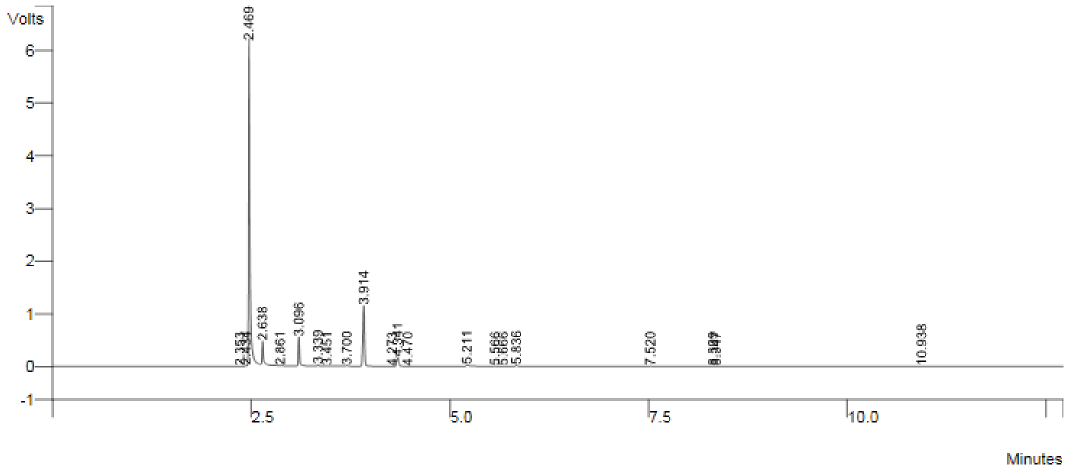

Figure S4. The GC traces of oxidation of acrolein catalyzed by Se-1-D-5-40.
